# Administration of a Multi-Genus Synbiotic to Broilers: Effects on Gut Health, Microbial Composition and Performance

**DOI:** 10.3390/ani13010113

**Published:** 2022-12-28

**Authors:** Zoi Prentza, Francesco Castellone, Matteo Legnardi, Birgit Antlinger, Maia Segura-Wang, Giorgos Kefalas, Nikolaos Papaioannou, Ioanna Stylianaki, Vasileios G. Papatsiros, Giovanni Franzo, Mattia Cecchinato, Konstantinos Koutoulis

**Affiliations:** 1 Department of Poultry Diseases, Faculty of Veterinary Science, School of Health Sciences, University of Thessaly, 43100 Karditsa, Greece; 2 DSM Nutritional Product UK, Heanor Gate Industrial Estate, Heanor DE75 7QZ, UK; 3 Department of Animal Medicine, Production and Health (MAPS), University of Padua, 35020 Legnaro, Italy; 4 DSM-BIOMIN Research Center, Technopark 1, 3430 Tulln, Austria; 5 NUEVO S.A., 32009 Schimatari, Greece; 6 Department of Pathology, School of Veterinary Medicine, Faculty of Health Sciences, Aristotle University of Thessaloniki, 54124 Thessaloniki, Greece; 7 Clinic of Medicine, Faculty of Veterinary Medicine, School of Health Sciences, University of Thessaly, 43100 Karditsa, Greece

**Keywords:** synbiotic, broilers, gut health, histopathology, microbiota

## Abstract

**Simple Summary:**

Since the use of antibiotics as growth promoters has been banned, the poultry sector is searching for alternatives to support production efficiency. Synbiotics, which consist of a mixture of prebiotics and probiotics, appear to be a promising way to do so by sustaining chickens’ gut health. In this study, the synbiotic PoultryStar^®^ sol was tested on three broiler flocks, reared in separate farms under typical field conditions. Compared to control chickens, those fed with the synbiotic throughout the productive cycle generally exhibited less histopathological lesions and had higher villi at intestinal level, and ultimately performed better in terms of body weight gain, feed conversion efficiency and liveability. The composition of the caecal microbial ecosystem was also studied, proving that synbiotic supplementation influenced the abundance of several bacterial populations. To fully understand the exact implications of these changes, further studies are required, which will be informed and facilitated by the present data.

**Abstract:**

In recent years, the applicability of prebiotics, probiotics and their mixtures, defined as synbiotics, in poultry production has received considerable attention. Following the increasing regulation of antibiotic use, these nutraceuticals are seen as an alternative way to sustain production efficiency and resistance to pathogens and stressors by modulating birds’ gut health. The aim of this study was to evaluate the benefits provided under field conditions by administering the multi-species synbiotic PoultryStar^®^ sol to broilers in drinking water. To this purpose, three Ross 308 broiler flocks, representing separate progenies of a breeder flock which was treated with the same synbiotic, were housed in separate farms, divided into treatment and control groups, and followed throughout the productive cycle. Synbiotic administration was shown to improve gut health even in absence of a challenge, with limited changes in terms of macroscopic intestinal lesions and more overt differences related to histopathological scores and villi length. Synbiotic-fed chickens performed consistently better in terms of body weight gain, feed conversion ratio and survivability. Lastly, the evaluation of the caecal microbiome through next-generation sequencing highlighted the effects of synbiotic supplementation on the composition of the bacterial population, the implications of which will, however, require further studies to be better comprehended.

## 1. Introduction

For decades, antibiotics have been utilized in the poultry industry to prevent and treat diseases and promote growth. Despite the significant improvements in technology and hygienic practices made at all stages of poultry production, bacterial diseases remain a persistent threat not only to animal health, but also to humans, with recent reports showing that *Salmonella* spp. and *Campylobacter* spp. are the most common causes of human foodborne bacterial diseases linked to poultry [1,2]. Due to the increasing awareness towards antimicrobial resistance, stricter regulations have been imposed on antibiotic use, and antibiotic-free poultry production has grown more and more popular. Nonetheless, several issues related to food safety and chicken welfare still need to be addressed to ensure the viability of such production systems, and there is an increasing need for alternative strategies to support production efficiency [3].

Since antibiotic growth promoters (AGPs) were crucial to controlling dysbacteriosis and enteropathogens [4], novel alternatives must be found following their ban to support gut health, which has several implications for the birds’ overall health, production efficiency, food safety and environmental impact [5]. Promoting eubiosis and minimizing enteric diseases is therefore essential to ensure the sustainability of poultry production. Several feed additives have been explored as natural alternatives to AGPs, including probiotics, prebiotics, synbiotics, organic acids, essential oils, enzymes, immunostimulants and phytobiotics [6]. Many of these products demonstrated beneficial effects similar to antibiotics in modulating the gut microbiome and improving the health and growth of the animals [7].

The studies conducted on synbiotics, which rely on a synergism between probiotics and prebiotics, have shown particularly promising results. The supplementation of different synbiotics to broilers was demonstrated to improve body weight (BW) gain and feed efficiency [8], reduce mortality [9], increase the resistance to heat stress [10], stimulate the development of the gut-associated lymphoid tissue (GALT) [11] and decrease the intestinal and carcass load of coliforms [12], *Clostridium perfringens* [13], *Campylobacter* spp. [14] and *Salmonella* spp. [15].

The benefits of synbiotics in terms of performance and intestinal health, along with their modulatory action on the microbial enteric composition [16], appear therefore well documented. Nonetheless, different formulations may have diverse features and modes of action and require a dedicated assessment of their efficacy and safety, including the risk of carrying antimicrobial resistance and producing deleterious metabolites [17]. In the present study, a commercial multi-species synbiotic product was administered to three broiler flocks, which were progenies of a breeder flock treated with the same synbiotic and were reared in three separate farms in typical field conditions, evaluating its effects on productive performance, gut health and caecal microbiota.

## 2. Materials and Methods

### 2.1. Experimental Setup

The present study was conducted on the broiler progenies of a broiler breeder flock that was treated with the synbiotic product PoultryStar^®^ sol (PS) (BIOMIN GmbH, Herzogenburg, Austria), as detailed in Prentza et al. [18]. Day-old chicks from eggs laid at 30, 35 and 40 weeks of age were placed into three different commercial farms (named 1, 2 and 3) and raised under typical field conditions until slaughter at 42 days of age (doa). In each farm, the chicks were divided between two different houses, observing a stocking density of 15 birds/m^2^. PS was administered in one of the two, while the other acted as control. In detail, depending on the house size, 8160 chicks were housed in the treatment house of farm 1, while 14,280 were placed in the control house; for farm 2, 11,730 and 10,200 birds were set up in the treatment and control houses, respectively; in farm 3, 15,198 chicks were placed in each of the houses.

### 2.2. Management

To ensure flock health and welfare and achieve good flock performance, management conditions followed the official guidelines for broiler birds [19]. Wheat straw and rice hulls were used as litter materials in cleaned and disinfected houses. The basal diet was formulated using maize, wheat and soybean meal in accordance with the official genetic line guidelines [20]. Feed and water were provided *ad libitum*. The lighting program started at 7 days of age, providing 4 hours of darkness and 20 hours of light. Birds were vaccinated against infectious bursal disease (IBD), infectious bronchitis (IB) and Newcastle disease (ND) at the hatchery following the local vaccination program.

### 2.3. Synbiotic Administration

PS, which is a synbiotic containing patented probiotic strains plus prebiotic fructooligosaccharides, was administered in one of the two houses in each of the three broiler farms, based on the manufacturer’s guidance. Specifically, a daily dosage of 20 g/1000 birds was administered in clean drinking water consecutively for the first three days of age, as recommended following the chicks’ placement, and then once a week till slaughter age was reached.

### 2.4. Bacterial Enteritis (BE) Scoring

To evaluate the chickens’ intestinal health and the presence of dysbiosis, the integrity of the intestinal wall was visually evaluated at three different time points (10, 28 and 38 doa) on ten birds randomly picked from different points of each house of each farm by applying a macroscopic lesion scoring system consisting of ten different parameters, which were scored 0 when absent and 1 when present. The individual scores were summed and divided by 2.5, yielding a total score ranging between 0 and 4 [21,22].

### 2.5. Histology

Specimens from different intestinal tract segments were collected at 38 doa from ten birds randomly picked from different points of each house of each farm for histopathological and morphometrical evaluations. In detail, 3 cm long segments were collected from the duodenum, jejunum, ileum and caecum, keeping the collection sites consistent for each tract, and placed in 10% neutral buffered formalin as described by Hoerr [23]. One mm thick transversal sections were cut after 48 hours, then sections of 3–5 μm were taken, stained with hematoxylin and eosin and evaluated. The histopathological scoring system proposed by Kraieski et al. [24] was adopted to assess the degree of inflammation in each section, grading the severity of the lesions on a 0–3 scale (0: absent or rare leukocytic infiltration; 1: leukocytic infiltration up to 5% of a ×400 field; 2: approximately 25% leukocytic infiltration of a ×400 field; 3: leukocytic infiltration in the range of 50% or more of a ×400 field).

The morphometry of the intestinal villi and crypts were examined in each section, performing optical capture and measurement with Image Pro-Plus v.6.0 software (Media Cybernetics, Silver Spring, MD, USA). The selection of the villi followed the criteria proposed by Gava et al. [25], namely the embedment of the base into the submucosa, the absence of any discontinuity or folding in the length of the villus, and the presence of intact epithelium at the tip.

### 2.6. Recording of Performance Parameters

To evaluate potential growth differences, 100 randomly selected chickens from each house of each farm were weighed longitudinally from 0 to 30 doa. The final BW was recorded when birds were loaded onto the trucks at 42 doa, and the feed conversion ratio (FCR) was calculated by dividing feed intake by the total BW gain. In addition, the carcass weight of 100 randomly selected birds from each group was measured at the slaughterhouse. Mortality was recorded daily throughout the whole cycle.

### 2.7. Evaluation of Enteric Microbiota

To evaluate the caecal microbial composition, Next Generation Sequencing (NGS) was performed on 60 caecal content samples taken at 38 doa from 10 randomly selected birds of each of the two houses of the three farms. The analyses, conducted using an Illumina MiSeq System (Illumina, San Diego, CA, USA) at LGC Genomics GmBH (Berlin, Germany), targeted the V3 region of the 16s rRNA gene, generating 2 × 300 paired-end sequences. Following a preliminary quality evaluation with FastQC 0.11.9, the forward and reverse sequences were trimmed at 195 bp and 220 bp, guaranteeing a minimal Phred score of 28 and allowing for a maximum expected error of 2 bases for each read. DADA2 [26] was used to infer the best fitting Amplicon Sequence Variants (ASVs), then the forward and reverse sequences were merged, and chimeric sequences were discarded. Finally, ASVs were converted to taxa using the SILVA 138 database [27,28] as a reference.

Alpha diversity was evaluated using the Simpson, Shannon, Chao1, and Observed species indexes. Permutational ANOVAs on the euclidean distances among samples were performed for significance testing between groups, after verifying that group dispersions were adequately homogeneous using the *betadisper* function of the *vegan* R package [29]. The absence of systematic biases was also confirmed by calculating the Spearman correlation between the treatment effect and all the other variables. Finally, the isolated treatment effect, along with the potential effect of other factors, was assessed by performing a differential abundance analysis with DESeq2.

### 2.8. Statistical Analyses

The existence of significant differences in terms of BE score, histopathological lesion score, villi and crypts height which may have been ascribed to treatment, farm effect or sampling age (in case of longitudinal sampling) were investigated using the non-parametric Kruskal–Wallis test followed by post-hoc Mann–Whitney test with Bonferroni correction. The treatment and farm effects on the carcass weight was investigated with a two-way ANOVA followed by post-hoc Tukey’s test. Log-rank test was used to compare the Kaplan–Meier survival curves with the *survival* package. All statistical analyses were performed in R (version 3.3.2) [30] setting the significance level to *p* < 0.05, with the sole exception of the differential abundance analysis of microbial populations, for which the significance level was set to *p* < 0.01.

## 3. Results

### 3.1. Bacterial Enteritis and Histopathological Lesion Scores

**Figure 1:**
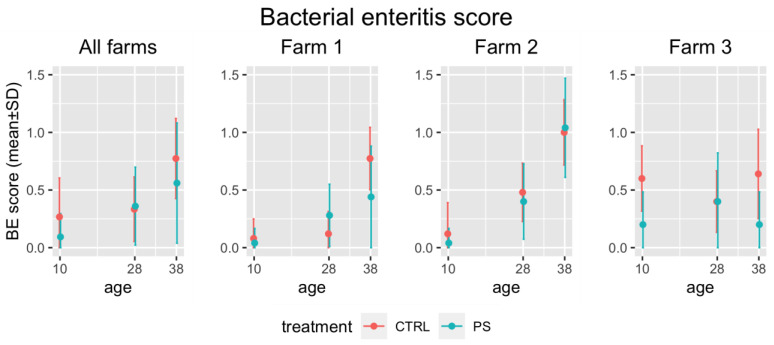
BE score measured in treatment (PS) and control (CTRL) groups at different time points by considering all farms together and then each one separately. BE scores are expressed on a scale from 0 (normal gastrointestinal tract) to 4 (severe dysbacteriosis).

The results obtained in terms of BE score (Figure 1) showed that the macroscopic signs of dysbacteriosis were limited in all treatment and control groups, whose scores were always below or around 1 on a scale from 0 to 4, where 0 corresponds to a normal gastrointestinal tract and 4 to a status of severe dysbacteriosis. However, the BE score was shown to increase significantly with age (*p* < 0.0001). Significant differences between PS-fed and control birds were found at 10 (*p* = 0.0228) and 38 doa (*p* = 0.0495) and when considering all ages together (*p* = 0.0162). Since the farm effect was also shown to be significant (*p* = 0.009), each farm was also assessed individually. The differences between synbiotic-treated and control groups were found to be limited to farm 3, where they were again significant at 10 (*p* = 0.0077) and 38 doa (*p* = 0.0108) and when considering all ages together (*p* = 0.0009). No statistically significant differences were observed in farm 1 and 2.

More overt differences were found in terms of histopathological lesions scores (Table 1). The average scores measured in each group mostly corresponded to a mild to moderate grade of inflammation in all intestinal tracts, with treated birds scoring significantly better in most of the comparisons. Significant between-farm differences were found at jejunum level (*p* = 0.0023), with farm 2 scoring worse than both farm 1 (*p* = 0.0005) and farm 3 (*p* = 0.04265), and at caecum level (*p* = 0.01499), which seemed mostly ascribable to differences between farm 2 and farm 3 (*p* = 0.03127).

### 3.2. Evaluation of Intestinal Villi and Crypts

The average villi and crypts lengths measured at 38 doa in PS-treated and control flocks, along with the villi/crypts (V/C) ratio, are shown in Table 2. Significant differences in villi length were observed in most of the enteric tracts, both at overall level and when considering each farm separately. On the other hand, the differences in terms of crypt length appeared more limited (Table 2). The farm effect proved significant for both villi (*p* < 0.0001 for all intestinal tracts) and crypts (*p* < 0.00001 for all intestinal tracts) length.

### 3.3. Performance Parameters

**Figure 2:**
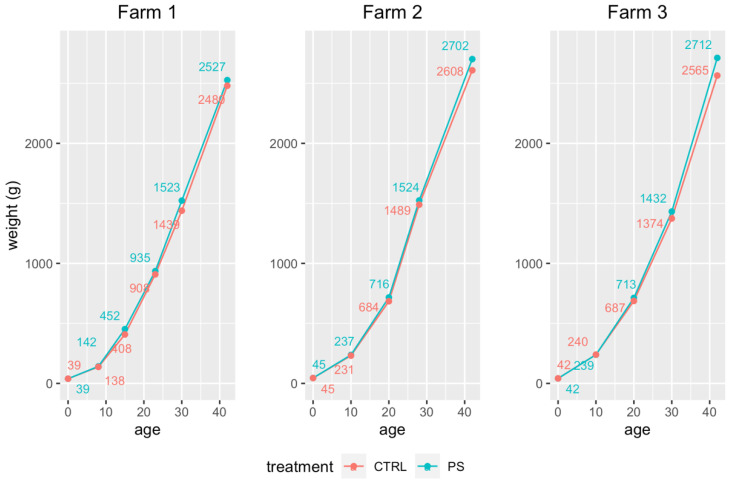
Live BW progression in synbiotic-treated (PS) and control chickens (CTRL).

Throughout the cycle, the average live BW measured in synbiotic-treated flocks was consistently higher than in control ones (Figure 2). The comparison of average carcass weights and FCRs, shown in Table 3, proves a significant treatment effect at overall level (*p* < 0.0001) and in each individual farm, while the farm effect and the interaction between treatment and farm effect were not.

**Figure 3:**
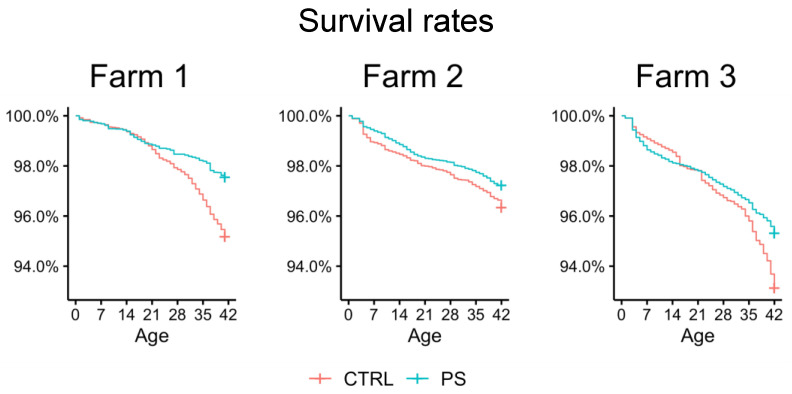
Kaplan–Meier survival estimates showing the mortality rates observed throughout the productive cycle in treated (PS) and control (CTRL) groups of each of the three farms.

Mortality rates observed in treated and control birds at overall level were 3.5% and 5.3%, respectively (*p* < 0.0001). Since the farm effect was found to be significant (*p* < 0.0001), each farm was also considered separately. In detail, mortality was 4.8% in the control group and 2.5% among treated birds in farm 1 (*p* < 0.0001); 3.7% and 2.8% in farm 2 (*p* = 0.0002); 6.9% and 4.7% in farm 3 (*p* < 0.0001) (Figure 3).

### 3.4. Evaluation of Enteric Microbiota

**Figure 4:**
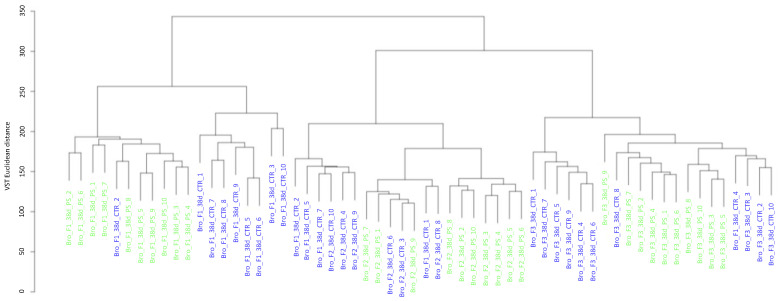
Dendrogram of caecal content samples, clustered on the euclidean distance between their taxonomic count data. Samples are color-coded by treatment (green for synbiotic-treated chickens, blue for control ones).

As shown in the dendrogram based on euclidean distances provided in Figure 4, samples clustered according to the farm in which they were collected. Samples from farm 2, which housed the progeny from eggs from 35-week-old layers, clustered more closely with farm 3 (progeny from 40-week-old layers) than with farm 1 (progeny from 30 weeks-old layers). Within farms, samples tended to cluster according to treatment, albeit with a few exceptions.

**Figure 5:**
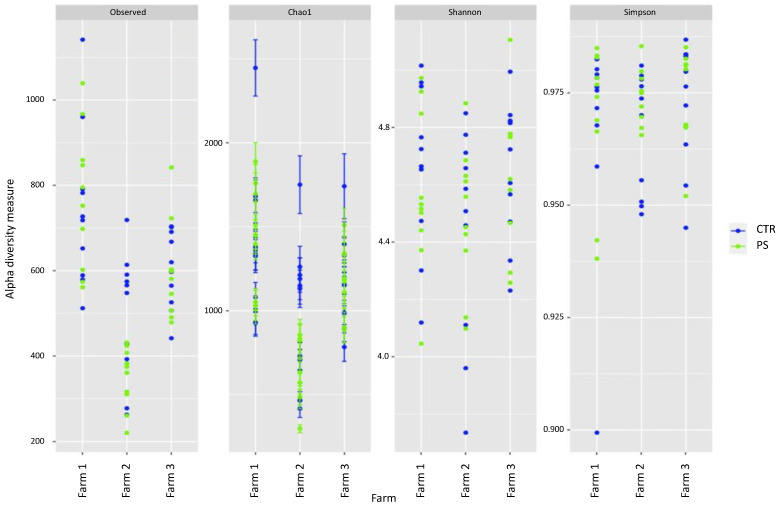
Alpha diversity measures for all caecal content samples divided by farm and color-coded based on treatment (green for synbiotic-treated chickens, blue for control ones).

The differences between treatments within the same farm was also made visible by some of the alpha diversity measures, namely the Observed species and the Chao1 indexes, although were less evident according to the Shannon and Simpson indexes (Figure 5). The effect appeared particularly noticeable in samples from farm 2, with synbiotic-treated chickens showing a lower species richness than control birds.

**Figure 6:**
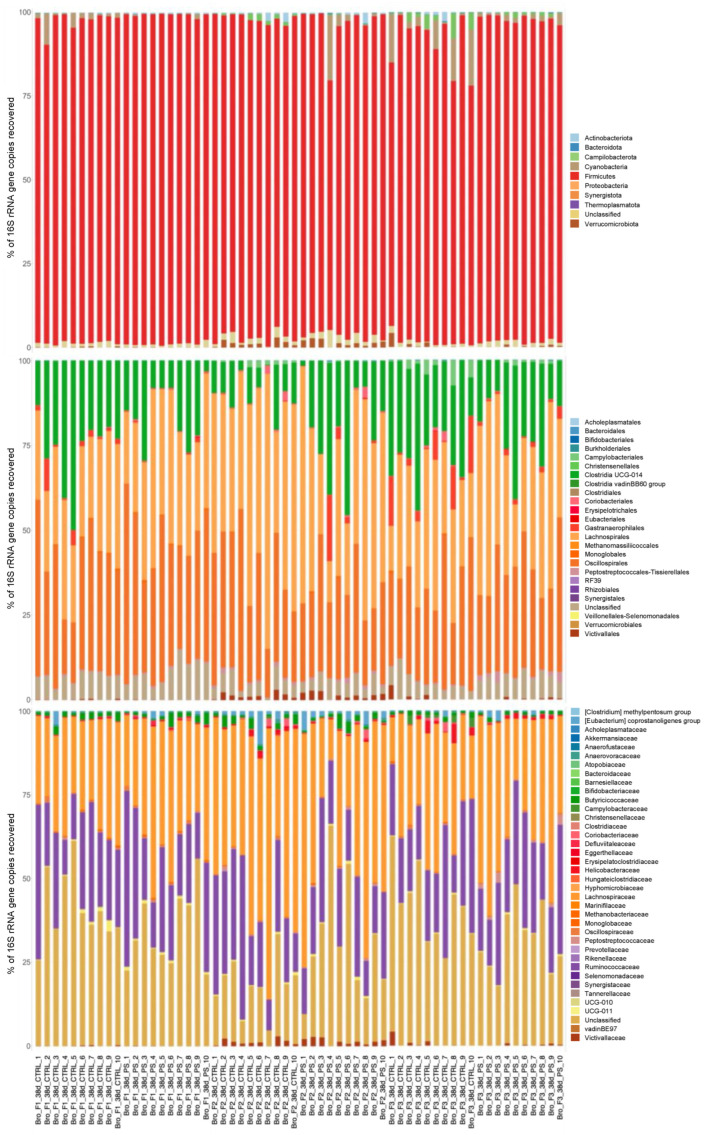
Relative microbial composition measured in individual caecal content samples, shown at Phylum (**top**), Order (**centre**) and Family (**bottom**) level.

A more comprehensive overview of diversity is provided by Figure 6, which shows the bacterial composition of each sample split on phylum, order and family level.

Since the beta dispersion within treatment group proved adequately homogeneous (*p* = 0.228) a permutational ANOVA test was subsequently performed, revealing siginificant differences between synbiotic-treated and control chickens at overall level (*p* = 0.002) and in each of the three farms (*p* < 0.001 in all three cases).

**Figure 7:**
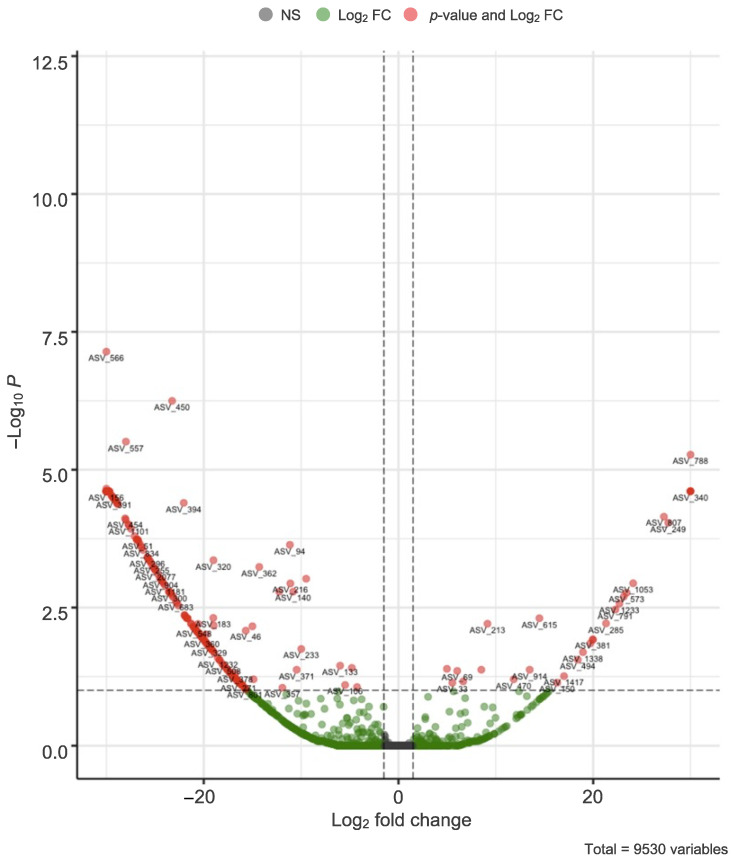
Volcano plot showing the differential abundance of ASVs due to the treatment effect. The statistical significance value was set to *p*-value < 0.01 (horizontal line), while, to be considered biologically significant, the effect size in terms of Fold Change (FC) should have had an absolute value higher than 3 (vertical lines at log_2_FC = 1.5).

Intercorrelation analysis revealed a significant Spearman correlation between the treatment and the length of caecal villi (ρ = −0.6195; *p* < 0.0001), but not with any other considered parameter. Considering the design of the study, these results allowed us to isolate the effect of the synbiotic treatment on bacterial composition. Out of 9530 ASVs, 65 had a significant differential abundance on an alpha level of 0.01 (after Benjamini–Hochberg multiple testing correction). By setting out the obtained adjusted *p*-values for each ASV against the respective fold changes (Figure 7), 40 ASVs were shown to be less abundant in synbiotic-treated than in control chickens, while 25 ASVs were overrepresented. The top 10 ASVs with the lowest adjusted *p*-values are shown in Table 4.

## 4. Discussion

The evaluation of macroscopic intestinal features and histological measurements allowed us to assess the chickens’ gut health, and how it was impacted by the synbiotic treatment. Although the BE and histopathological lesion scores suggested good intestinal health in all treatment and control groups, likely due to the absence of a challenge, some differences in favor of the treated chickens could still be noted. In particular, significantly lower BE scores were observed in synbiotic-fed birds only in farm 3, while more overt differences were found in terms of histopathological lesions in most intestinal tracts of chickens raised in all farms. These results were in line with those observed after administering PS to the group of broiler breeders that birthed the investigated progenies, which also pointed at a general intestinal eubiosis in both treatment and control groups, with the former scoring better overall [18].

The BE scores measured in this study allowed us to demonstrate a significant age effect, showing a biologically plausible increase in subsequent time points. In addition, between-farm differences were present according to both scores. Such an effect, which was observed also for other evaluated parameters, could easily be ascribed to environmental factors related to the individual farms, such as differences in management, stockmanship, housing, farm location and others. These variations should be considered inherent to the field conditions in which the study was conducted to reproduce the real-life application of the tested synbiotic.

The evaluation of villi and crypts length at 38 doa provided additional insights on the effect of PS. Longer villi are considered an indicator of a greater surface area and thus a greater adsorption capability [31,32], while shorter villi and deeper crypts may lead to poor nutrient absorption, increased secretions in the gastrointestinal tract, and poorer performance [33]. Reduced villi length also appears to be associated to macroscopic lesions suggestive of dysbacteriosis [22]. The villi of the treated broilers were significantly longer across most intestinal tracts and in all three farms, in agreement with other studies which reported the same beneficial effect for other synbiotics [34,35,36,37,38]. On the other hand, the differences in terms of crypt depth were less marked and consistent. Conflicting evidence on the effect of synbiotic supplementation on intestinal crypts may also be found in the literature, with different formulations leading to an increase [36], decrease [39] or not affecting their depth [15]. Trends similar to the present study were found in the previous experiment conducted on breeders [18], consolidating the knowledge about the effects of PS administration on gut morphology.

By promoting good intestinal health and preventing any undesired condition of dysbacteriosis or inflammation, synbiotic administration should ultimately promote production efficiency. In this study, synbiotic-treated flocks performed significantly better according to several parameters, including feed conversion ratio, carcass BW and daily cumulative mortality rate. These results agree with several other studies in which other synbiotics were tested [8,37,40,41,42], and further support the promising application of synbiotics to poultry production.

The impact of PS administration on caecal bacteriome was also investigated through high-throughput sequencing. Caeca represent the enteric tract with the highest microbial density, mainly composed of obligate anaerobes belonging to the phyla Firmicutes and Bacteroidetes [43]. The studied flocks showed a large predominance of Firmicutes, particularly members of the class Clostridia, as expected in broilers [44,45]. In comparison, the microbial composition of the broiler breeders which birthed the broiler progenies used for this study was similarly dominated by Firmicutes but, coherently with the older age at sampling, the overall diversity was higher [18].

A clear treatment effect was visible in all farms, significantly impacting the abundance of 65 ASVs. Among the most influenced were representatives of *Lachnospiraceae*, a family of cellulolytic bacteria capable of metabolizing non-starch polysaccharides that are among the earliest colonizers of the caecum of broiler chickens [46,47], which were overrepresented in synbiotic-fed flocks compared to control ones. On the other hand, ASVs belonging to the genera *Faecalibacterium*, which are common inhabitants of the caeca involved in butyrate production and in the anti-inflammatory response [48,49], and *Monoglobus*, a less abundant component of the intestinal microbiome capable of degrading pectin [50], were underrepresented, along with others classifiable as *Clostridia UCG-014*. Overall, based on alpha diversity measures, PS chickens exhibited a lower species richness than control ones.

Considering the complexity and variability of the gut microbiota, it is hard to ascertain the implications of the observed changes. An increasing number of studies on synbiotics are using molecular assays to investigate their effects on gut bacterial populations, reporting different results. For instance, Baffoni et al. [14] and Pineda-Quiroga et al. [51] found that, similar to the present study, species richness was decreased by synbiotic treatment, but the opposite was reported by other authors [52,53]. The taxa affected by synbiotic supplementation were widely variable [51,52,54], further complicating the interpretation of these findings. This diversity can be easily motivated by considering the many variables ascribable to environmental, nutritional, and host factors [16] as well as to the formulation and administration protocol of different synbiotic products. Nonetheless, these data are still valuable to obtain a more complete picture of the mechanism through which a specific synbiotic acts, and also add to the existing general knowledge on synbiotics and nutraceuticals for future comparisons.

## 5. Conclusions

The presented results support the benefits achieved by the administration of PoultryStar^®^ sol in broilers, which improved the productive performance in terms of FCR, carcass weight and mortality rate. Treated chickens also exhibited a better intestinal health, having lower histopathological lesion scores and longer villi across most intestinal tracts. In addition, synbiotic supplementation was shown to influence the caecal microbial ecosystem, causing some taxa to be more or less abundant in synbiotic-fed flocks. The exact implications of these changes will, however, require further studies to be better understood.

## Figures and Tables

**Table 1:** Mean ± standard deviation of the histopathological lesion scores measured at duodenum, jejunum, ileum and caecum level of treated (PS) and control (CTRL) birds. Scores are reported on a scale from 0 (absent or rare leukocytic infiltration) to 3 (leukocytic infiltration in the range of 50% of a ×400 field). *p*-values below 0.05, marking the statistical significance of a difference observed when comparing treatment and control groups across all farms or in each farm individually, are underlined.

Farm	Intestinal Tract	Histopathological Lesion Score		
		CTRL	PS	*p*-Value
All farms	Duodenum	1.667 ± 0.480	1.367 ± 0.490	0.0211
	Jejunum	1.833 ± 0.379	1.3 ± 0.466	<0.0001
	Ileum	1.8 ± 0.407	1.233 ± 0.430	<0.0001
	Caecum	2.067 ± 0.691	1.5 ± 0.630	0.0081
Farm 1	Duodenum	1 ± 0.000	1.6 ± 0.516	0.005
	Jejunum	1.5 ± 0.527	1.1 ± 0.316	0.06362
	Ileum	1.5 ± 0.527	1 ± 0.000	0.0137
	Caecum	2.3 ± 0.675	1.2 ± 0.483	0.0017
Farm 2	Duodenum	2 ± 0.000	1.3 ± 0.483	0.0016
	Jejunum	2 ± 0.000	1.7 ± 0.483	0.0767
	Ileum	1.9 ± 0.316	1.4 ± 0.516	0.0251
	Caecum	1.6 ± 0.516	1.5 ± 0.527	0.6934
Farm 3	Duodenum	2 ± 0.000	1.2 ± 0.133	0.0004
	Jejunum	2 ± 0.000	1.1 ± 0.100	<0.0001
	Ileum	2 ± 0.000	1.3 ± 0.157	0.0016
	Caecum	2.3 ± 0.213	1.8 ± 0.249	0.1557

**Table 2:** Mean ± standard deviation of villi and crypts length (measured in μm) in each intestinal tract of synbiotic-treated (PS) and control (CTRL) birds. *p*-values below 0.05, marking the statistical significance of a difference observed when comparing treatment and control groups across all farms or in each farm individually, are underlined.

Farm	Intestinal Tract	Villi Length			Crypt Length		
		CTRL	PS	*p*-Value	CTRL	PS	*p*-Value
All farms	Duodenum	725.7 ± 150.2	760.6 ± 125.6	0.0221	160.8 ± 62.7	155.9 ± 70.2	0.1343
	Jejunum	446.7 ± 117.0	498.0 ± 109.0	<0.0001	114.8 ± 50.0	121.9 ± 57.0	0.5289
	Ileum	255.7 ± 90.2	304.4 ± 85.9	<0.0001	107.2 ± 55.6	109.4 ± 43.6	0.2507
	Caecum	114.3 ± 46.2	146.0 ± 81.8	0.0113	91.7 ± 34.7	94.0 ± 38.1	0.764
Farm 1	Duodenum	709.8 ± 136.2	710.9 ± 178.7	0.9478	216.4 ± 73.1	234.9 ± 63.1	0.2385
	Jejunum	518.4 ± 94.8	525.6 ± 98.0	0.6969	165.1 ± 53.2	184.4 ± 50.4	0.0589
	Ileum	345.3 ± 64.6	382.9 ± 94.0	0.046	158.4 ± 67.9	163.2 ± 29.9	0.0189
	Caecum	152.0 ± 42.2	224.3 ± 93.4	0.0002	123.1 ± 29.2	132.2 ± 37.3	0.2482
Farm 2	Duodenum	791.6 ± 109.9	804.0 ± 72.3	0.8795	147.4 ± 31.0	118.3 ± 28.4	<0.0001
	Jejunum	344.0 ± 87.9	484.6 ± 92.1	<0.0001	88.5 ± 18.9	88.2 ± 22.3	0.5372
	Ileum	199.0 ± 46.5	276.9 ± 40.3	<0.0001	80.5 ± 17.8	82.5 ± 13.9	0.5259
	Caecum	78.8 ± 29.4	112.3 ± 31.9	<0.0001	81.2 ± 23.7	70.6 ± 19.8	0.0329
Farm 3	Duodenum	659.7 ± 170.2	766.8 ± 79.4	0.0025	118.7 ± 22.4	114.6 ± 24.8	0.6075
	Jejunum	477.8 ± 88.9	483.8 ± 129.9	0.5837	90.87 ± 23.3	93.0 ± 29.0	0.855
	Ileum	222.6 ± 76.3	254.3 ± 49.9	0.0383	82.9 ± 21.5	82.3 ± 16.1	0.9341
	Caecum	112.1 ± 33.2	101.5 ± 34.3	0.0926	70.5 ± 25.7	79.5 ± 19.1	0.0141

**Table 3:** Average carcass weights and feed conversion ratios (FCRs) measured in treated and control houses of the three farms. *p*-values below 0.05, marking the statistical significance of a difference observed when comparing treatment and control groups, are underlined.

Farm	Average Carcass Weight (FCR)		
	CTRL	PS	*p*-Value
Farm 1	1948 g (1.85)	2021 g (1.79)	0.0094
Farm 2	2001 g (1.70)	2095 g (1.64)	0.0052
Farm 3	1979 g (1.76)	2087 g (1.70)	0.0079

**Table 4:** Top ten differentially abundant ASVs for the treatment effect based on the adjusted *p*-value. The direction of differential abundance is indicated by the sign of the Log_2_ Fold Change.

ASV	Log2 Fold Change	Standard Error	Adjusted *p*-Value	Lowest Resolved Taxon
ASV_566	−30.000000	4.494941	2.4864 × 10^−11^	Faecalibacterium
ASV_450	−23.252888	3.715454	3.8889 × 10^−10^	Monoglobus
ASV_557	−27.994668	4.727891	3.1965 × 10^−9^	Clostridia UCG-014
ASV_788	30.000000	5.187864	7.3500 × 10^−9^	Lachnospiraceae
ASV_326	−30.000000	5.454703	3.8013 × 10^−8^	Clostridia UCG-014
ASV_156	−30.000000	5.827878	2.6374 × 10^−7^	Clostridia UCG-014
ASV_159	−30.000000	5.839874	2.7902 × 10^−7^	Clostridia UCG-014
ASV_275	−29.839008	5.840154	3.2338 × 10^−7^	Clostridia UCG-014
ASV_340	30.000000	5.832041	2.6895 × 10^−7^	Clostridia
ASV_395	−29.762335	5.834230	3.3727 × 10^−7^	Clostridia UCG-014

## Data Availability

Not applicable.
